# G = E: What GWAS Can Tell Us about the Environment

**DOI:** 10.1371/journal.pgen.1005765

**Published:** 2016-02-11

**Authors:** Suzanne H. Gage, George Davey Smith, Jennifer J. Ware, Jonathan Flint, Marcus R. Munafò

**Affiliations:** 1 MRC Integrative Epidemiology Unit (IEU) at the University of Bristol, Bristol, United Kingdom; 2 UK Centre for Tobacco and Alcohol Studies, School of Experimental Psychology, University of Bristol, Bristol, United Kingdom; 3 School of Social and Community Medicine, University of Bristol, Bristol, United Kingdom; 4 Wellcome Trust Centre for Human Genetics, University of Oxford, Oxford, United Kingdom; Georgia Institute of Technology, UNITED STATES

## Abstract

As our understanding of genetics has improved, genome-wide association studies (GWAS) have identified numerous variants associated with lifestyle behaviours and health outcomes. However, what is sometimes overlooked is the possibility that genetic variants identified in GWAS of disease might reflect the effect of modifiable risk factors as well as direct genetic effects. We discuss this possibility with illustrative examples from tobacco and alcohol research, in which genetic variants that predict behavioural phenotypes have been seen in GWAS of diseases known to be causally related to these behaviours. This consideration has implications for the interpretation of GWAS findings.

## Introduction

The rapid growth in genome-wide association studies (GWAS) has resulted in the identification of common genetic variants associated with behavioural traits, from biomarker phenotypes that capture the downstream consequences of behaviour (e.g., body mass index [BMI]) [1] to the behaviours themselves (e.g., tobacco and alcohol use) [2]. While the success of GWAS has generated insights into the biological mechanisms underpinning these traits (see Box 1), it is less appreciated that it has also begun to tell us about the causal effects of modifiable or environmental influences on these traits. For example, a genetic variant at a locus containing the *NPC1L1* gene is strongly associated with low-density lipoprotein (LDL) cholesterol level as well as with the risk of cardiovascular events. This is not because *NPC1L1* is independently associated with cardiovascular problems, but simply because high cholesterol is a causal risk factor for the disease [3–5]. In other words, there are a number of cases where GWAS of disease outcomes have identified loci that capture modifiable risk factors rather than direct biological pathways. Here, we explain how this insight can inform the interpretation of GWAS results.

**Box 1. *CHRNA5-A3-B4* and Cigarette Smoking**

The most robust finding to emerge from GWAS of smoking phenotypes is the association between the nicotinic receptor gene cluster *CHRNA5-A3-B4* on chromosome 15 (at 15q25) and smoking quantity. This gene cluster encodes three nicotinic acetylcholine receptor subunit proteins: alpha-5, alpha-3, and beta-4. An association between the nonsynonymous variant rs16969968 in *CHRNA5* and nicotine dependence was first reported in 2007 in a candidate gene study [39], with the minor allele found to confer increased risk. The following year, the same locus (tagged by rs1051730 in *CHRNA3*, in high linkage disequilibrium with rs16969968) was found to be associated with smoking quantity, this time in a GWAS conducted by Thorgeirsson and colleagues [11]. This study also highlighted an association between rs1051730 and two smoking-related diseases, lung cancer and peripheral arterial disease. These initial findings renewed interest in *CHRNA5* and *CHRNA3* and the role played by these genes in nicotine dependence, and led on to a series of preclinical follow-up studies focused on determining the mechanism underlying the observed associations with smoking behaviour and disease. Functional research has demonstrated that the minor allele at rs16969968 (i.e., the risk variant for heavier smoking) is associated with a decreased maximal response to a nicotine agonist relative to the major allele in vitro [40]. Subsequent research using alpha-5 knockout mice has further illustrated the role that *CHRNA5* plays in determining response to nicotine. Using a nicotine self-administration paradigm, Fowler and colleagues [41] observed that knockout mice responded far more vigorously than wild-type mice for nicotine infusions at high doses and, unlike wild-types, did not self-titrate nicotine delivery. Deficient alpha-5 signalling attenuated the aversive effects of nicotine that would normally serve to limit its intake.

The association between the *CHRNA5-A3-B4* locus and smoking quantity [2,12,42–44], alongside other smoking-related phenotypes and diseases, has been replicated in numerous studies. GWAS of lung cancer [14] and chronic obstructive pulmonary disease [15] have also identified this locus, and follow-up candidate gene studies have suggested a role in bladder cancer [45], emphysema [46], and upper aerodigestive tract cancer [47], diseases for which smoking has already been recognised as a causal factor [48]. Some of these findings were used to argue that there is an independent effect of this locus on the disease, given evidence of residual association between variant and disease following adjustment for self-reported smoking quantity. However, this is likely due to the imprecision of self-report measures of heaviness of smoking and misclassification of smoking status. Studies using biomarkers of tobacco exposure have illustrated that rs1051730/rs16969968 accounts for a far greater proportion of variance in nicotine metabolite levels relative to self-report measures of daily tobacco consumption [13,49,50]. When we use the per allele effect of rs1051730 on cotinine levels, for example, to estimate the association between genotype and lung cancer risk, this accords with published data, which supports the conclusion that the effect of this locus on lung cancer risk is mediated largely, if not wholly, via level of tobacco exposure [13].

## Genetics and Causal Inference

The relationship between phenocopy and genocopy (see Box 2) lies at the heart of the Mendelian randomization approach, which seeks to leverage genetic information to identify causal relationships between modifiable exposures and disease outcomes. The principles of Mendelian randomization have been described in detail elsewhere [6–8]. In brief, genetic variants are used as proxies (i.e., instrumental variables) for modifiable exposures. If the assumptions of Mendelian randomization hold, these proxies should not be associated with the factors that confound observational associations and will not be subject to reverse causation. This has become an increasingly popular technique for establishing whether an observational association between an exposure and an outcome is likely to be causal. However, while Mendelian randomization makes use of information obtained (principally) via GWAS, our argument is that the same reasoning can directly inform our interpretation of GWAS results.

**Box 2. Genocopy and Phenocopy**

The term “phenocopy” is attributed to Goldschmidt [51] and describes the situation where an environmental effect results in the same effect as that produced by a genetic variant. It is generally used to describe diseases that are similar to some genetic syndrome but that can also be caused by environmental exposures. The term “genocopy,” attributed to Schmalhausen [52], is essentially the reverse of phenocopy and describes the situation in which a genetic variant produces an outcome that could equally be produced by an environmental exposure. The critical point is that genetic and environmental causes of disease can be seen as essentially equivalent; as Goldschmidt wrote in 1938, “different causes produce the same end effect, presumably by changing the same developmental processes in an identical way” [51]. More recently, Zuckerkandl and Villet have argued that “no doubt all environmental effects can be mimicked by one or several mutations,” again suggesting that genetic and environmental influences can be regarded as both equivalent and interchangeable [53].

Implementing Mendelian randomization techniques can be challenging, principally because single genetic variants (and even polygenic risk scores) typically capture only a small proportion of variance in the exposure of interest. An ideal instrument would exactly mimic the exposure of interest without being associated with confounding variables, but this is, of course, impossible in practice. Genetic variants are, therefore, generally weak instruments, meaning that very large sample sizes are required to attain adequate statistical power. Historically, datasets were required with information on genotype, outcome, and exposure of interest in order to run such studies. Methodological developments now allow the application of Mendelian randomization across two different samples if no single sample is available that includes data on genotype, the exposure, and the outcome.

**Fig 1:**
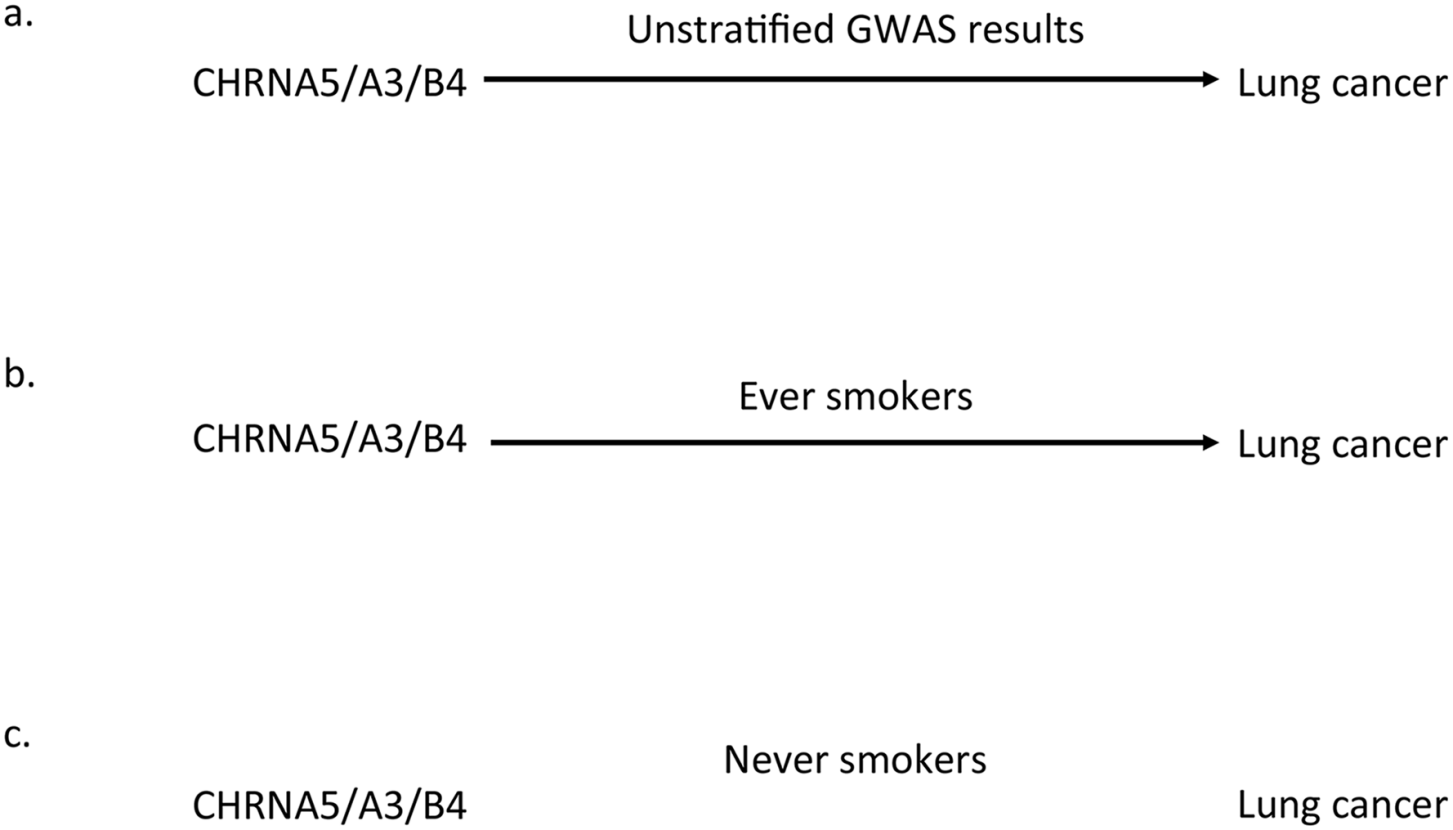
Illustration of the Mendelian randomization framework. In Mendelian randomization, if there is a causal effect of the exposure (e.g., smoking heaviness) that is being captured by the genotype on the outcome (e.g., lung cancer), then an association of genotype with the outcome should be detectable in a sufficiently large unstratified GWAS (panel A). This can be confirmed in a stratified analysis, where an association of genotype with the outcome should only be seen in the exposed group (i.e., smokers, panel B) and not the unexposed group (i.e., never-smokers, panel C). This is a special case of gene × environment (G × E) interaction, where both G and E are known, although it will not always be possible to stratify on the exposure, and stratification (which can be considered a form of statistical adjustment) can introduce other potential biases in certain circumstances (see Box 3).

Conventional Mendelian randomization uses genetic markers known to be associated with a modifiable exposure of interest, for which there is also a known observational association between the modifiable exposure and an outcome of interest. In an ideal situation, the association of the genotype with the outcome can be tested across strata of individuals who are positive or negative for the putative mediating exposure (e.g., ever-smokers versus never-smokers). If there is a causal effect of the exposure (e.g., smoking heaviness) that is being captured by the genotype, then an association of the genotype with the outcome should only be seen in the exposed group and not the unexposed group (see Fig 1) [9]. This is a special case of gene × environment (G × E) interaction, where both G and E are known, although it will not always be possible to stratify on the exposure, and stratification (which can be considered a form of statistical adjustment) can introduce other potential biases in certain circumstances (see Box 3) [10]. Nevertheless, if differences in the magnitude of association observed in exposed and unexposed groups are very large, this is convincing evidence of a causal pathway via the exposure. Therefore, a logical extension of Mendelian randomization is that any GWAS will, in principle, identify exposures that are causally associated with the outcome of interest in that GWAS.

**Box 3. Stratification in Mendelian Randomization**

For behavioural exposures such as tobacco or alcohol use, stratification on the exposure of interest can be a powerful means of testing the pleiotropy assumption that is central to Mendelian randomization. While endogenous exposures (e.g., cholesterol levels) can never be zero, only higher or lower in different individuals, behavioural exposures are generally limited to a subset of the population (e.g., smokers). In principle, a genetic variant associated with heaviness of smoking should be associated with an outcome of interest (e.g., BMI) only among those exposed to this putative causal agent (i.e., ever-smokers) and not those unexposed (i.e., never-smokers). Whether this association differs between strata can be assessed using an interaction test.

**Fig 2:**
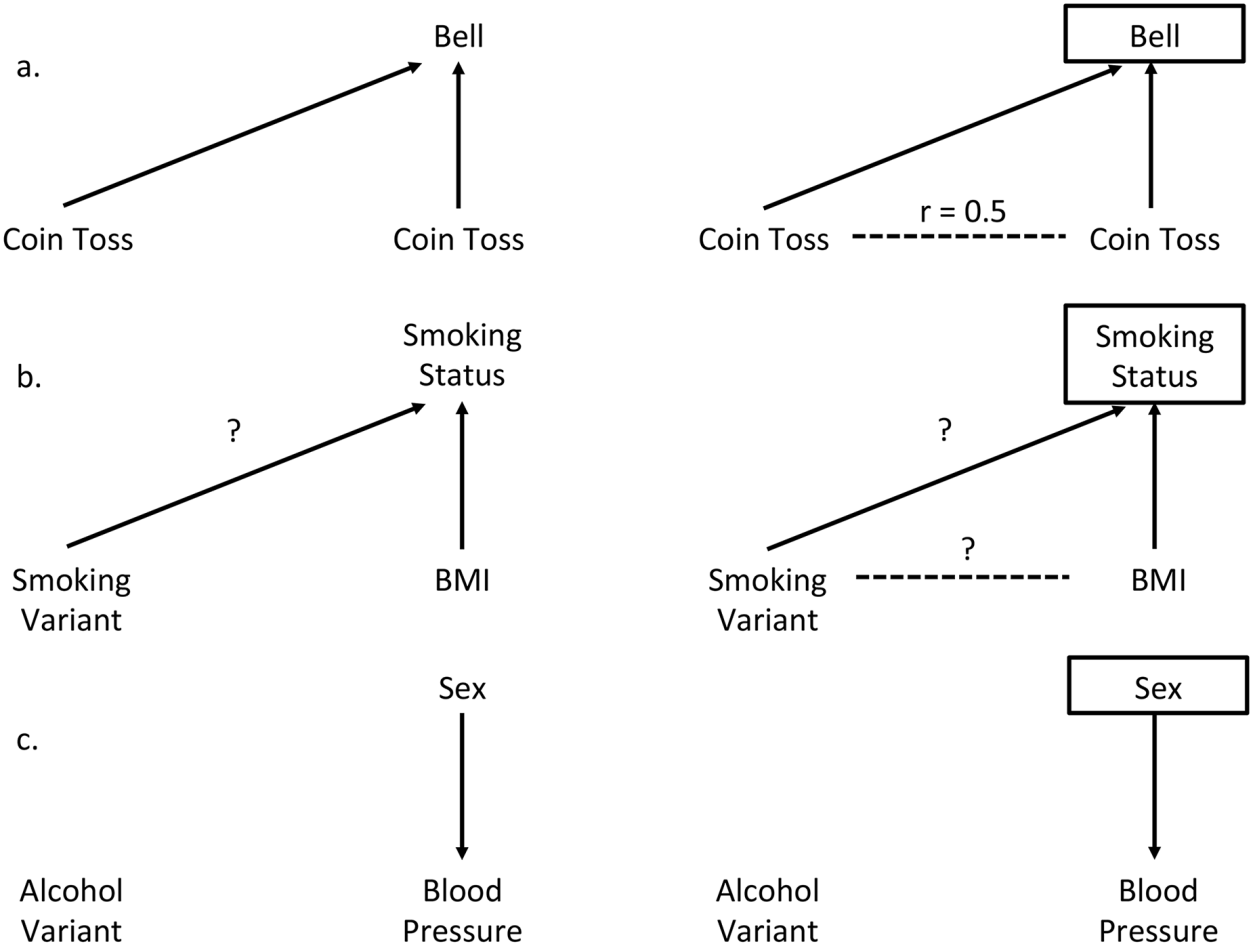
Illustration of collider bias. Panel A shows the basic premise of collider bias. In this example, a bell is sounded whenever either coin come up “heads.” The result of one coin toss is independent of the other. However, if we stratify on the bell ringing, seeing “heads” on both coins is not independent and a spurious correlation is induced. Panel B shows this with the example of stratifying on smoking status. If the variant used as an instrument for heaviness of smoking is also associated with smoking status (i.e., ever-smoker versus never-smoker), and if BMI also influences smoking status, then there is a risk of collider bias if we stratify on smoking status. Panel C shows an example where stratification will not introduce collider bias, as sex is not an effect of either possession of a genetic variant that predicts alcohol consumption or of blood pressure.

However, stratification on a common effect can introduce collider bias [10,54], which can result in a spurious correlation between otherwise independent variables (Fig 2A). In the case of the *CHRNA5-A3-B4* variants used in Mendelian randomization analyses of smoking, the assumption is that these variants are principally associated with heaviness of smoking rather than smoking status, in which case the risk of collider bias is reduced, as smoking initiation is not a common effect (Fig 2B). However, if these variants are shown to be associated with smoking initiation [55], this risk would be increased. Stratification does not always introduce the risk collider bias—for example, in a Mendelian randomization analysis of alcohol consumption and blood pressure, the analysis was stratified by participant sex due to differences in alcohol consumption among men and women in East Asian populations [23]. This does not introduce the possibility of collider bias because sex cannot be an effect of the genetic variant in question; sex is determined by a different genetic variation, which is inherited independently of other variants, and sex cannot be an effect of blood pressure (Fig 2C).

**Fig 3:**
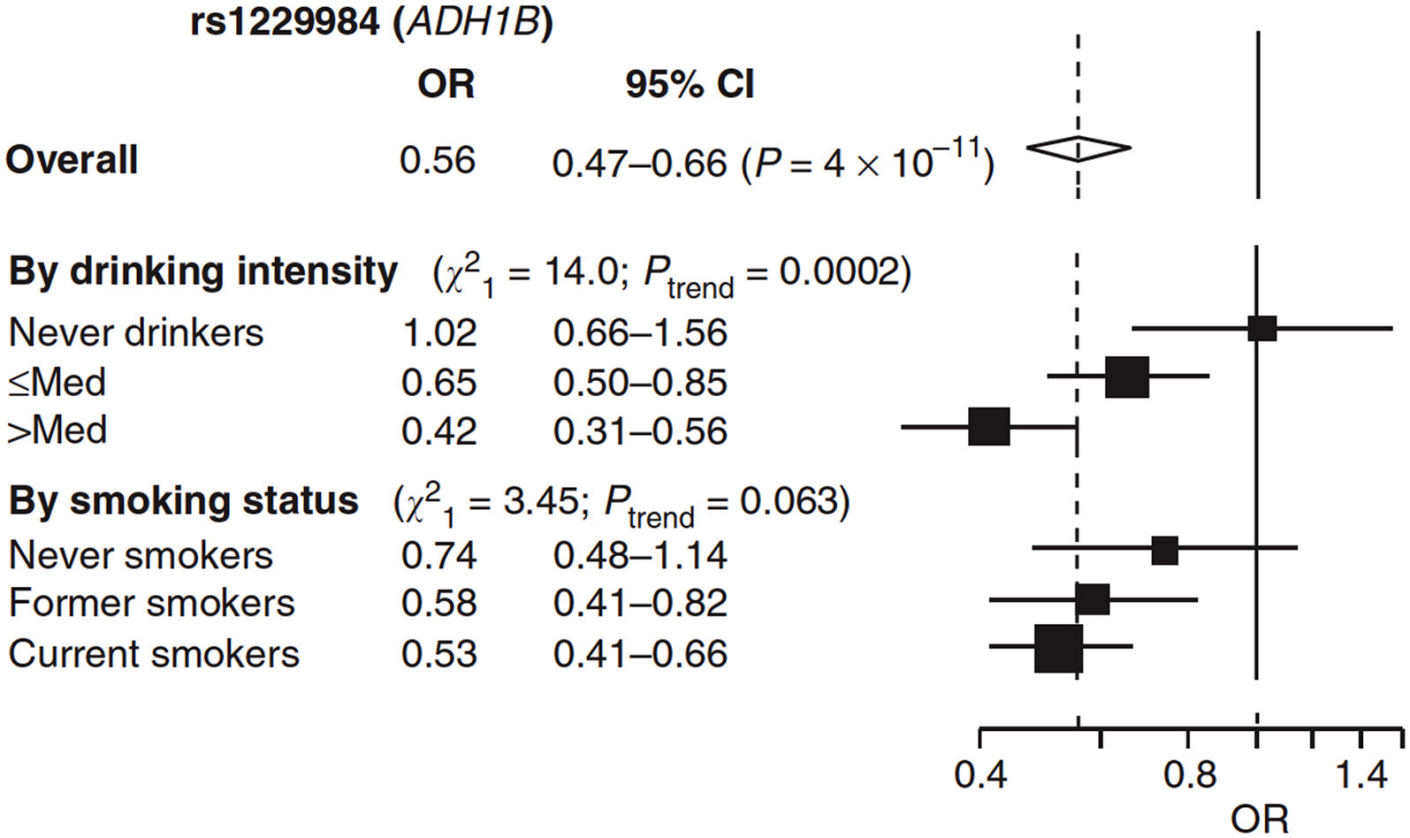
Association of *ADH1B* genotype with risk of upper aerodigestive cancer. Risk of upper aerodigestive cancer by *ADH1B* genetic variation, stratified by drinking intensity and smoking status, is shown as the odds ratio (OR) of upper aerodigestive cancer by re1229984 (*ADH1B*) genotype comparing rare allele (dominant model) carriers versus common allele homozygous genotype. ORs are standardised by age, sex, study centre, cumulative alcohol consumption, and, when relevant, smoking. ORs and 95% CI are derived from fixed effects models. *Figure adapted from Hashibe et al*. *(2008)* [57] *with permission granted by Nature Publishing Group*.

It is also worth remembering that the environmental exposure that is used for stratification is subject to the usual problems of confounding. Keller has argued that many gene × environment interaction studies do not adequately control for potential confounders because they do not include covariate × gene and covariate × environment interaction terms [56]. For example, *ADH1B* genotype shows clear association with risk of upper aerodigestive cancer among alcohol drinkers, but not non-drinkers, consistent with a causal effect of alcohol consumption (Fig 3) [14]. However, a similar (albeit weaker) pattern is observed when stratification is based on smoking status rather than alcohol consumption, because these exposures are correlated. In these cases, the interaction will be stronger for the causal factor (i.e., when stratification is based on drinking status rather than smoking status).

We illustrate the application of Mendelian randomization reasoning to GWAS data using the example of two behavioural phenotypes—tobacco and alcohol use. These are phenotypes for which GWAS has identified a number of associated loci. A number of disease outcomes are also known to be associated with tobacco and alcohol use, and therefore serve as proof of principle for our argument that GWAS can identify the effects of modifiable exposures.

## Cigarettes and Alcohol

The strongest genetic signal for tobacco-use phenotypes identified via GWAS is located in a gene cluster on chromosome 15 containing the *CHRNA5*, *CHRNA3*, and *CHRNB4* genes (*CHRNA5-A3-B4*), which encode the alpha-5, alpha-3, and beta-4 nicotinic acetylcholine receptor subunits, respectively. Each additional copy of the minor risk allele at this locus is associated with one extra cigarette smoked per day. The locus [11] accounts for approximately 1% of the variation in cigarette consumption in daily smokers [12] and approximately 4% of the variation in cotinine levels, the primary metabolite of nicotine and a more precise biomarker of exposure [13]. The same locus has been identified in GWAS of lung cancer [11,14], peripheral arterial disease [11], and chronic obstructive pulmonary disease [15]. One parsimonious interpretation of these results is that these are all diseases for which cigarette smoking is a strong, causal risk factor. Indeed, the effect of smoking on these outcomes is sufficiently strong that variants associated with heaviness of smoking achieve genome-wide significance even in unstratified GWAS (i.e., where smokers and never-smokers are not considered separately). When stratified, one should see the association only in ever-smokers and not in never-smokers (see Box 3) [16–18], although due to misclassification (i.e., misreporting of smoking status), this is not always the case.

A subtler example arises from the association of *ALDH2* with alcohol consumption. This gene encodes aldehyde dehydrogenase, an enzyme responsible for metabolizing acetaldehyde (a metabolite of alcohol) to acetic acid. When less of this enzyme is present, acetaldehyde can build up after alcohol consumption, leading to unpleasant side effects. Therefore, the minor allele is robustly associated with reduced alcohol consumption [19]. The frequency of the minor allele at the *ALDH2* locus is very low in samples of European ancestry but is relatively common in samples of East Asian ancestry. It is, therefore, only associated with alcohol consumption in the latter population. As a result, in GWAS of high blood pressure, *ALDH2* was not identified in studies that recruited predominantly European samples [20] but was identified in studies that recruited East Asian samples (once genotyping chips that adequately tagged the *ALDH2* locus were used) [21,22]. This confirms the results of Mendelian randomization analyses of alcohol consumption and blood pressure conducted prior to these later GWAS [23]. In other words, alcohol consumption causes high blood pressure, and this is detected in GWAS of high blood pressure, but only when tested in populations in which the variants associated with alcohol consumption are sufficiently common. This provides strong evidence that the identification of this locus in the GWAS is due to a causal effect of alcohol consumption rather than being due to shared genetic aetiology or to pleiotropy. Similarly, *ALDH2* has emerged in GWAS of esophageal cancer in East Asian samples [24], confirming earlier Mendelian randomization analyses [25].

Behavioural traits such as tobacco and alcohol use can be regarded as intermediate traits, which are under a degree of genetic influence but which are themselves direct causal agents influencing various health outcomes. However, a critical difference between these and more usual intermediate phenotypes (such as LDL cholesterol) is that whereas both may be direct causal agents and amenable to intervention for therapeutic benefit, the former may be entirely absent (i.e., non-smokers, non-drinkers), whereas the latter cannot be (i.e., no one has a cholesterol level of zero). Genetic variants may influence whether or not someone smokes or drinks, or how much they smoke or drink, or both.

## Implications

As GWAS of disease outcomes are carried out on increasingly large samples, more loci will be identified, promising to deliver insights into underlying biological mechanisms. However, as we have seen, it will become increasingly important to also consider whether these associations reflect effects of modifiable exposures. This will require the triangulation of evidence across GWAS of disease outcomes and GWAS of behavioural phenotypes to determine the cases in which signals identified for behavioural phenotypes are the same as those identified for disease phenotypes. Unfortunately, this approach is hampered at present by the relative lack of GWAS of behavioural phenotypes—while we have identified a number of variants associated with tobacco and (to a lesser extent) alcohol use, as well as obesity, this is not yet the case for exposures such as cannabis use. Nevertheless, this situation is rapidly changing—for example, there are now several variants that have been shown to be associated with caffeine consumption [26]. It is also worth noting that both the *CHRNA5-A3-B4* and *ALDH2* loci were initially identified in candidate gene studies.

Already, intriguing hints are emerging that larger GWAS are beginning to identify potential environmental or behavioural causes of disease. A recent GWAS led by the Psychiatric Genomics Consortium identified 108 loci associated with schizophrenia [27]. One locus that reached genome-wide significance is located in the *CHRNA5-A3-B4* gene cluster on chromosome 15, which, as we have seen, has been consistently shown to contain multiple loci strongly associated with heaviness of smoking [2] among cigarette smokers. There are two possible explanations for this finding. One is that there may be genetic variants in this region that independently influence both heaviness of smoking and schizophrenia risk (i.e., genetic pleiotropy). The other is that this signal captures a causal effect of cigarette smoking on schizophrenia (reflecting a dose–response relationship among the smokers in the study). Again, there is a precedent for this pattern of results: the same region was shown to be associated with lung cancer risk [11], but it is likely that this effect operates entirely via cigarette smoking [13].

Critically, while the identification of 108 loci associated with schizophrenia was rightly heralded as a breakthrough in our understanding of the genetic determinants of schizophrenia, very little was made of this potentially vital insight. If smoking is indeed a causal risk factor for schizophrenia, then this has immediate and dramatic implications for public health, prevention, and treatment. Intriguingly, since the publication of these results, several other studies have been published that also support a causal role for smoking in schizophrenia and psychosis [28–31]. It is notable that one study reports a stratified analysis that suggests an association of *CHRNA5-A3-B4* genotype with antipsychotic medication prescription (as a proxy of psychotic illness) in ever-smokers but not in never-smokers [32]. This is analogous to the case of *CHRNA5-A3-B4* genotype and lung cancer risk, although the evidence in relation to schizophrenia is currently only suggestive.

As GWAS of other behavioural phenotypes such as personality and intelligence emerge, it will be interesting to see whether variants known to influence tobacco or alcohol use emerge, given the strong observational associations between these phenotypes. At the same time, GWAS of other behavioural phenotypes such as cannabis use will in due course provide loci that may signal causal effects of these behaviours on a range of other outcomes (notably schizophrenia).

## Identifying Causal Pathways

For any locus identified via GWAS, we need to consider whether this reflects a potential modifiable risk factor. However, it is difficult to exclude the possibility that this locus is independently associated with both a modifiable risk factor and the disease outcome directly. For example, a recent study found an association between a polygenic risk score for schizophrenia (combining multiple variants identified with genome-wide significance into a single risk score) and cannabis use [32]. The authors concluded that this indicates that some of the association between schizophrenia and cannabis is due to a shared genetic aetiology. However, an alternative explanation could be that genetic predisposition to schizophrenia (and behaviours associated with this) increases the risk of cannabis use. Here, the distinction between mediated and biological pleiotropy is useful—the former refers to the genetic influence on the outcome operating via an exposure or intermediate phenotype, while the latter refers to a direct and independent genetic influence on both the exposure and the outcome [33]. Mediated pleiotropy is a single process leading to a cascade of downstream events, ultimately leading to a distal outcome. In this way, genetic variation at the *FTO* locus influences BMI and, in turn, blood pressure, hypertension, coronary heart disease, and so on [33]. While statistical adjustment (e.g., for BMI) can help dissect these pathways, this can be problematic where residual associations may exist due to measurement error, such as in the case of *CHRNA5-A3-B4*, smoking, and lung cancer risk [13]. Biological pleiotropy is more problematic and renders causal inference difficult.

A hierarchy of approaches supports stronger causal inference regarding the role of modifiable exposures on disease outcomes (see Table 1). Ultimately, what is required is a triangulation of evidence using these different approaches, ranging from whole genome methods to more focused analyses, to determine whether the results obtained using these different methods align consistently [34]. First, genetic correlation [35,36] can be used to identify shared genetic influences (e.g., cannabis use and schizophrenia). This approach allows all genotyped common variants to be interrogated, with correlations with modifiable exposures suggestive of possible causality. Second, conventional Mendelian randomization analyses (using single variants or polygenic risk scores) can be used to establish evidence that genetic proxies for a modifiable exposure of interest (e.g., cannabis use) associate with the outcome thought to be influenced by the exposure (e.g., schizophrenia) [33]. Single variant approaches are appropriate when the genetic variants play a known and relatively specific role in the pathway of interest (e.g., *ALDH2* and alcohol consumption), but these will capture a smaller proportion of the variance in the exposure than polygenic risk scores. Third, when adequate genetic variants have been identified for both the exposure and the outcome, bidirectional Mendelian randomization can be used to determine with greater confidence the likely direction of any causal relationship [33]. Fourth, a range of sensitivity analyses exist that can inform the interpretation of the findings, such as the extent to which (biological) pleiotropy has an impact on the causal estimates derived from conventional Mendelian randomization methods. This may be particularly relevant when polygenic risk scores comprising variants that act on a range of biological pathways are used. These methods include Mendelian randomization Egger regression [37] and the Kang median instrument approach [38]. Those relationships that survive this hierarchy of approaches are strong candidates for further interrogation in mechanistic or experimental studies.

**Table 1:** Hierarchy of evidence.

Strength of evidence (low > high)	Description
Genetic correlation	This method estimates genetic correlation using GWAS summary statistics, using properties of linkage disequilibrium to allow for rapid screening for correlations among a diverse set of traits without the need for individual level data. However, this approach is still subject to genetic confounding (pleiotropy) and misclassification bias and requires larger samples than methods that use individual data. A well-powered null finding would argue against a causal association between exposure and outcome. However, direction of causation cannot be identified.
Polygenic risk score association	Polygenic risk scores can be derived where there are multiple variants identified with genome-wide significance for a trait or disease. These can be weighted to represent the proportion of the variance in the risk factor that they explain, and used as a proxy for an exposure to investigate associations of interest. The use of a risk score allows for a larger proportion of the variance to be explained, although it is very likely it will increase the risk of pleiotropy.
Bidirectional Mendelian randomization with polygenic risk scores	If polygenic risk scores are available for both the exposure and outcome of interest, associations can be investigated in both directions, which may provide evidence in support of an association in a particular causal direction.
Mendelian randomization sensitivity analyses	Mendelian randomization Egger regression extends the basic Mendelian randomization method by meta-analysing the SNP outcome association from each individual SNP that is associated with the exposure. This treats each SNP as akin to a small study in a traditional meta-analysis. Regression analysis, allowing variation in the intercept, means it is able to provide an estimate of the extent to which genetic pleiotropy has an impact on the causal estimates from Mendelian randomization analyses. Kang median instrument analysis has been shown to identify causal effects as long as fewer than 50% of instruments are invalid, without requiring knowledge of which instruments are invalid. It also allows identification of when this 50% threshold is violated.

The approaches described here can also be informative with respect to null results. If a modifiable exposure is under genetic influence and is also causally related to a disease outcome, we would expect to eventually see genetic variants associated with the exposure emerge in a GWAS of the outcome, given sufficient sample size. If this is not seen, this suggests that there may be no causal pathway operating (or that any causal relationship is very weak). Of course, interpreting null results must be done cautiously, particularly in cases where the prevalence of the modifiable exposure or the minor allele frequency differs across populations. Current GWAS cannot control for these sources of heterogeneity, which may impact the power of GWAS to identify modifiable exposures in the way we have described. Cross-contextual comparisons (e.g., across GWAS conducted in different populations) may be informative in these cases.

## Conclusion

As we run larger and larger GWAS, some of the signals that emerge may turn out to reflect the action of modifiable (e.g., environmental or behavioural) exposures, rather than more direct biological effects. At present, what is likely to be required to understand these pathways is a two-step approach in which initial GWAS findings are interrogated further in studies in which detailed phenotype information is available. At present, this is not always possible—for example, a lack of smoking status information in the studies contributing to the recent schizophrenia GWAS means it is not possible to test the possible causal effect of smoking in a stratified analysis. However, as large, richly phenotyped cohort studies (e.g., UK Biobank) emerge, it will become possible to identify modifiable exposures from genetic data and to dissect those pathways within the same cohort. Here, “modifiable” can refer to substance use, but also to factors such as cholesterol or metabolite levels or blood pressure, which are directly influenced by lifestyle choices. A failure to appreciate this point will hamper our ability to translate the results of GWAS into health benefits, by focusing attention on possible biological pathways when, in fact, the target for intervention could be a modifiable environmental or behavioural exposure. We also need to be cautious when using statistical adjustment to test whether a genetic variant operates entirely via the supposed intermediate behavioural pathway. Sometimes, the most parsimonious explanation (e.g., smoking causes lung cancer) is the best one.

## References

[1] Speliotes EK, Willer CJ, Berndt SI, Monda KL, Thorleifsson G, Jackson AU, et al Association analyses of 249,796 individuals reveal 18 new loci associated with body mass index. Nature Genetics. 2010;42:937–948. 10.1038/ng.686 20935630 PMC3014648

[2] Tobacco-and-Genetics-Consortium. Genome-wide meta-analyses identify multiple loci associated with smoking behavior. Nature Genetics. 2010;42:441–447. 10.1038/ng.571 20418890 PMC2914600

[3] Cannon CP, Blazing MA, Giugliano RP, McCagg A, White JA, Theroux P, et al Ezetimibe Added to Statin Therapy after Acute Coronary Syndromes. New England Journal of Medicine. 2015;372:2387–2397. 10.1056/NEJMoa1410489 26039521

[4] Ference BA, Majeed F, Penumetcha R, Flack JM, Brook RD. Effect of naturally random allocation to lower low-density lipoprotein cholesterol on the risk of coronary heart disease mediated by polymorphisms in NPC1L1, HMGCR, or both: a 2 x 2 factorial mendelian randomization study. Journal of the American College of Cardiology. 2015;65:1552–1561. 10.1016/j.jacc.2015.02.020 25770315 PMC6101243

[5] Jarcho JA, Keaney JF Jr,. Proof That Lower Is Better—LDL Cholesterol and IMPROVE-IT. New England Journal of Medicine. 2015;372:2448–2450. 10.1056/NEJMe1507041 26039520

[6] Davey Smith G, Ebrahim S. 'Mendelian randomization': can genetic epidemiology contribute to understanding environmental determinants of disease? International Journal of Epidemiology. 2003;32:1–22. 12689998 10.1093/ije/dyg070

[7] Davey Smith G, Ebrahim S. What can Mendelian randomisation tell us about modifiable behavioural and environmental exposures? BMJ. 2005;330:1076–1079. 15879400 10.1136/bmj.330.7499.1076 PMC557238

[8] Davey Smith G, Lawlor DA, Harbord R, Timpson N, Day I, Ebrahim S. Clustered environments and randomized genes: a fundamental distinction between conventional and genetic epidemiology. PloS Med. 2007;4:e352 18076282 10.1371/journal.pmed.0040352 PMC2121108

[9] Davey Smith G. Use of genetic markers and gene-diet interactions for interrogating population-level causal influences of diet on health. Genes and Nutrition. 2011;6:27–43. 10.1007/s12263-010-0181-y 21437028 PMC3040803

[10] Cole SR, Platt RW, Schisterman EF, Chu H, Westreich D, Richardson D, et al Illustrating bias due to conditioning on a collider. International Journal of Epidemiology. 2010;39:417–420. 10.1093/ije/dyp334 19926667 PMC2846442

[11] Thorgeirsson TE, Geller F, Sulem P, Rafnar T, Wiste A, Magnusson KP, et al A variant associated with nicotine dependence, lung cancer and peripheral arterial disease. Nature. 2008;452:638–642. 10.1038/nature06846 18385739 PMC4539558

[12] Ware JJ, van den Bree MB, Munafo MR. Association of the CHRNA5-A3-B4 gene cluster with heaviness of smoking: a meta-analysis. Nicotine & Tobacco Research. 2011;13:1167–1175. 22071378 10.1093/ntr/ntr118 PMC3223575

[13] Munafo MR, Timofeeva MN, Morris RW, Prieto-Merino D, Sattar N, Brennan P, et al Association between genetic variants on chromosome 15q25 locus and objective measures of tobacco exposure. Journal of the National Cancer Institute. 2012;104:740–748. 10.1093/jnci/djs191 22534784 PMC3352832

[14] Amos CI, Wu X, Broderick P, Gorlov IP, Gu J, Eisen T, et al Genome-wide association scan of tag SNPs identifies a susceptibility locus for lung cancer at 15q25.1. Nature Genetics. 2008;40:616–622. 10.1038/ng.109 18385676 PMC2713680

[15] Pillai SG, Ge D, Zhu G, Kong X, Shianna KV, Need AC, et al A genome-wide association study in chronic obstructive pulmonary disease (COPD): identification of two major susceptibility loci. PLoS Genet. 2009;5:e1000421 10.1371/journal.pgen.1000421 19300482 PMC2650282

[16] Wang Y, Broderick P, Matakidou A, Eisen T, Houlston RS. Chromosome 15q25 (CHRNA3-CHRNA5) variation impacts indirectly on lung cancer risk. PLoS ONE. 2011;6:e19085 10.1371/journal.pone.0019085 21559498 PMC3084737

[17] Timofeeva MN, Hung RJ, Rafnar T, Christiani DC, Field JK, Bickeboller H, et al Influence of common genetic variation on lung cancer risk: meta-analysis of 14 900 cases and 29 485 controls. Human Molecular Genetics. 2012;21:4980–4395. 10.1093/hmg/dds334 22899653 PMC3607485

[18] Gabrielsen ME, Romundstad P, Langhammer A, Krokan HE, Skorpen F. Association between a 15q25 gene variant, nicotine-related habits, lung cancer and COPD among 56,307 individuals from the HUNT study in Norway. European Journal of Human Genetics. 2013;21:1293–1299. 10.1038/ejhg.2013.26 23443019 PMC3798835

[19] Luczak SE, Glatt SJ, Wall TL. Meta-analyses of ALDH2 and ADH1B with alcohol dependence in Asians. Psychological Bulletin. 2006;132:607–621. 16822169 10.1037/0033-2909.132.4.607

[20] International Consortium for Blood Pressure Genome-Wide Association S, Ehret GB, Munroe PB, Rice KM, Bochud M, Johnson AD, et al Genetic variants in novel pathways influence blood pressure and cardiovascular disease risk. Nature. 2011;478:103–109. 10.1038/nature10405 21909115 PMC3340926

[21] Kato N, Takeuchi F, Tabara Y, Kelly TN, Go MJ, Sim X, et al Meta-analysis of genome-wide association studies identifies common variants associated with blood pressure variation in east Asians. Nature Genetics. 2011;43:531–538. 10.1038/ng.834 21572416 PMC3158568

[22] Lu X, Wang L, Lin X, Huang J, Charles Gu C, He M, et al Genome-wide association study in Chinese identifies novel loci for blood pressure and hypertension. Human Molecular Genetics. 2015;24:865–874. 10.1093/hmg/ddu478 25249183 PMC4303798

[23] Chen L, Davey Smith G, Harbord RM, Lewis SJ. Alcohol intake and blood pressure: a systematic review implementing a Mendelian randomization approach. PLoS Med. 2008;5:e52 10.1371/journal.pmed.0050052 18318597 PMC2265305

[24] Wu C, Kraft P, Zhai K, Chang J, Wang Z, Li Y, et al Genome-wide association analyses of esophageal squamous cell carcinoma in Chinese identify multiple susceptibility loci and gene-environment interactions. Nature Genetics. 2012;44:1090–1097. 10.1038/ng.2411 22960999

[25] Lewis SJ, Davey Smith G. Alcohol, ALDH2, and esophageal cancer: a meta-analysis which illustrates the potentials and limitations of a Mendelian randomization approach. Cancer Epidemiology, Biomarkers & Prevention. 2005;14:1967–1971. 10.1158/1055-9965.EPI-05-0196 16103445

[26] Cornelis MC, Monda KL, Yu K, Paynter N, Azzato EM, Bennett SN, et al Genome-wide meta-analysis identifies regions on 7p21 (AHR) and 15q24 (CYP1A2) as determinants of habitual caffeine consumption. PLoS Genetics. 2011;7:e1002033 10.1371/journal.pgen.1002033 21490707 PMC3071630

[27] Schizophrenia-Working-Group-of-the-Psychiatric-Genomics-Consortium. Biological insights from 108 schizophrenia-associated genetic loci. Nature. 2014;511:421–427. 10.1038/nature13595 25056061 PMC4112379

[28] Gurillo P, Jauhar S, Murray R, MacCabe JH. Does tobacco use cause psychosis? Systematic review and meta-analysis. Lancet Psychiatry. 2015;2:718–725. 10.1016/S2215-0366(15)00152-2 26249303 PMC4698800

[29] Kendler KS, Lonn SL, Sundquist J, Sundquist K. Smoking and schizophrenia in population cohorts of Swedish women and men: A prospective co-relative control study. American Journal of Psychiatry. 2015; 117(11):1092–1100. 10.1176/appi.ajp.2015.15010126 PMC4651774 26046339

[30] McGrath J, Alati R, Clavarino A, Williams G, Bor W, Najman J, et al Age at first tobacco use and risk of subsequent psychosis-related outcomes: A birth cohort study. Australian and New Zealand Journal of Psychiatry. 2015 E-pub ahead of print. 10.1177/0004867415587341 25991762

[31] Wium-Andersen MK, Orsted DD, Nordestgaard BG. Tobacco smoking is causally associated with antipsychotic medication use and schizophrenia, but not with antidepressant medication use or depression. International journal of Epidemiology. 2015; 44(2):566–577. 10.1093/ije/dyv090 26054357

[32] Power RA, Verweij KJ, Zuhair M, Montgomery GW, Henders AK, Heath AC, et al Genetic predisposition to schizophrenia associated with increased use of cannabis. Molecular Psychiatry. 2014;19:1201–1204. 10.1038/mp.2014.51 24957864 PMC4382963

[33] Davey Smith G, Hemani G. Mendelian randomization: genetic anchors for causal inference in epidemiological studies. Human Molecular Genetics. 2014;23:R89–98. 10.1093/hmg/ddu328 25064373 PMC4170722

[34] Gage SH, Munafo MR, Davey Smith G. Causal inference in Developmental Origins of Health and Disease (DOHaD) research. Annual Review of Psychology. 2015;67:567–85. 10.1146/annurev-psych-122414-033352 26442667

[35] Bulik-Sullivan B, Finucane HK, Anttila V, Gusev A, Day FR, Consortium R, et al An atlas of genetic correlations across human diseases and traits. bioRxiv. 2015:47(11);1236–41. 10.1038/ng.3406 PMC4797329 26414676

[36] Pickrell J, Berisa T, Segurel L, Tung JY, Hinds D. Detection and interpretation of shared genetic influences on 40 human traits. bioRxiv. 2015 10.1101/019885 http://biorxiv.org/content/early/2015/05/27/019885 PMC5207801 27182965

[37] Bowden J, Davey Smith G, Burgess S. Mendelian randomization with invalid instruments: effect estimation and bias detection through Egger regression. International journal of epidemiology. 2015;44:512–525. 10.1093/ije/dyv080 26050253 PMC4469799

[38] Kang H, Zhang A, Cai TT, Small DS. Instrumental variables estimation with some invalid instruments and its application to Mendelian randomization. Journal of the American Statistical Association. 2015 E-pub ahead of print. http://arxiv.org/abs/1401.5755

[39] Saccone SF, Hinrichs AL, Saccone NL, Chase GA, Konvicka K, Madden PA, et al Cholinergic nicotinic receptor genes implicated in a nicotine dependence association study targeting 348 candidate genes with 3713 SNPs. Human Molecular Genetics. 2007;16:36–49. 17135278 10.1093/hmg/ddl438 PMC2270437

[40] Bierut LJ, Stitzel JA, Wang JC, Hinrichs AL, Grucza RA, Xuei X, et al Variants in nicotinic receptors and risk for nicotine dependence. American Journal of Psychiatry. 2008;165:1163–1171. 10.1176/appi.ajp.2008.07111711 18519524 PMC2574742

[41] Fowler CD, Lu Q, Johnson PM, Marks MJ, Kenny PJ. Habenular alpha5 nicotinic receptor subunit signalling controls nicotine intake. Nature. 2011;471:597–601. 10.1038/nature09797 21278726 PMC3079537

[42] Thorgeirsson TE, Gudbjartsson DF, Surakka I, Vink JM, Amin N, Geller F, et al Sequence variants at CHRNB3-CHRNA6 and CYP2A6 affect smoking behavior. Nature Genetics. 2010;42:448–453. 10.1038/ng.573 20418888 PMC3080600

[43] David SP, Hamidovic A, Chen GK, Bergen AW, Wessel J, Kasberger JL, et al Genome-wide meta-analyses of smoking behaviors in African Americans. Translational Psychiatry. 2012;2:e119. 10.1038/tp.2012.41 PMC3365260 22832964

[44] Liu JZ, Tozzi F, Waterworth DM, Pillai SG, Muglia P, Middleton L, et al Meta-analysis and imputation refines the association of 15q25 with smoking quantity. Nature Genetics. 2010;42:436–440. 10.1038/ng.572 20418889 PMC3612983

[45] Kaur-Knudsen D, Bojesen SE, Tybjaerg-Hansen A, Nordestgaard BG. Nicotinic acetylcholine receptor polymorphism, smoking behavior, and tobacco-related cancer and lung and cardiovascular diseases: a cohort study. Journal of Clinical Oncology. 2011;29:2875–2882. 10.1200/JCO.2010.32.9870 21646606

[46] Lambrechts D, Buysschaert I, Zanen P, Coolen J, Lays N, Cuppens H, et al The 15q24/25 susceptibility variant for lung cancer and chronic obstructive pulmonary disease is associated with emphysema. American Journal of Respiratory and Critical Care Medicine. 2010;181:486–493. 10.1164/rccm.200909-1364OC 20007924

[47] Lips EH, Gaborieau V, McKay JD, Chabrier A, Hung RJ, Boffetta P, et al Association between a 15q25 gene variant, smoking quantity and tobacco-related cancers among 17 000 individuals. International Journal of Epidemiology. 2010;39:563–577. 10.1093/ije/dyp288 19776245 PMC2913450

[48] U.S.-Department-of-Health-and-Human-Services. The health consequences of smoking: A report of the Surgeon General National Center for Chronic, Disease Prevention and Health Promotion. Atlanta, GA 2004.

[49] Le Marchand L, Derby KS, Murphy SE, Hecht SS, Hatsukami D, Carmella SG, et al Smokers with the CHRNA lung cancer-associated variants are exposed to higher levels of nicotine equivalents and a carcinogenic tobacco-specific nitrosamine. Cancer Research. 2008;68:9137–9140. 10.1158/0008-5472.CAN-08-2271 19010884 PMC2587068

[50] Keskitalo K, Broms U, Heliovaara M, Ripatti S, Surakka I, Perola M, et al Association of serum cotinine level with a cluster of three nicotinic acetylcholine receptor genes (CHRNA3/CHRNA5/CHRNB4) on chromosome 15. Human Molecular Genetics. 2009;18:4007–4012. 10.1093/hmg/ddp322 19628476 PMC2748889

[51] Goldschmidt RB. Physiological Genetics. New York: McGraw-Hill Book Company Inc; 1938.

[52] Gause GF. The relation of adaptability to adaptation. Quarterly Review of Biology. 1942;17:99–114.

[53] Zuckerkandl E, Villet R. Concentration-affinity equivalence in gene regulation: convergence of genetic and environmental effects. Proceedings of the National Academy of Sciences USA. 1988;85:4784–4788. 10.1073/pnas.85.13.4784 PMC280520 3387439

[54] Glymour MM, Tchetgen Tchetgen EJ, Robins JM. Credible Mendelian randomization studies: approaches for evaluating the instrumental variable assumptions. American Journal of Epidemiology. 2012;175:332–339. 10.1093/aje/kwr323 22247045 PMC3366596

[55] Taylor AE, Munafo MR, CARTA Consortium. Commentary: Does mortality from smoking have implications for future Mendelian randomization studies? International Journal of Epidemiology. 2014;43:1483–1486. 10.1093/ije/dyu151 25125581 PMC4190520

[56] Keller MC. Gene x environment interaction studies have not properly controlled for potential confounders: the problem and the (simple) solution. Biological Psychiatry. 2014;75:18–24. 10.1016/j.biopsych.2013.09.006 24135711 PMC3859520

[57] Hashibe M, McKay JD, Curado MP, Oliveira JC, Koifman S, Koifman R, et al Multiple ADH genes are associated with upper aerodigestive cancers. Nature Genetics. 2008;40:707–709. 10.1038/ng.151 18500343

