# Correction: G = E: What GWAS Can Tell Us about the Environment

**DOI:** 10.1371/journal.pgen.1006065

**Published:** 2016-05-12

**Authors:** Suzanne H. Gage, George Davey Smith, Jennifer J. Ware, Jonathan Flint, Marcus R. Munafò

There is an error in Fig 2. The correlation coefficient in panel a should be negative. The authors have provided a corrected figure and legend here.

**Fig 2:**
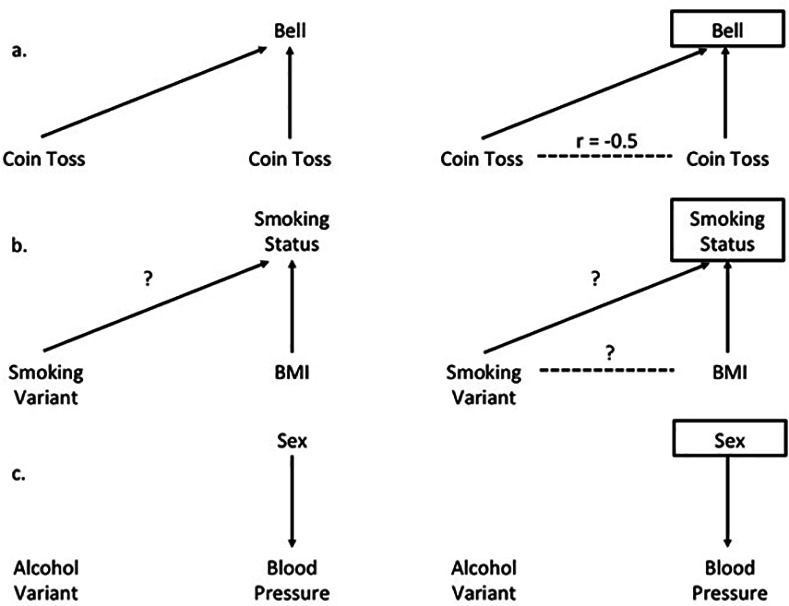
Illustration of Collider Bias. Panel a shows the basic premise of collider bias. In this example, a bell is sounded whenever either coin come up ‘heads’. The result of one coin toss is independent of the other. However, if we stratify on the bell ringing, seeing ‘heads’ on both coins is not independent and a spurious inverse correlation induced. Panel b shows this with the example of stratifying on smoking status. If the variant used as an instrument for heaviness of smoking is also associated with smoking status (i.e., ever vs never smoker), and if BMI also influences smoking status, then there is a risk of collider bias if we stratify on smoking status. Panel c shows an example where stratification will not introduce collider bias, as sex is not an effect of either possession of a genetic variant that predicts alcohol consumption, or of blood pressure.
